# Deep learning-based automated speech detection as a marker of social functioning in late-life depression

**DOI:** 10.1017/S0033291719003994

**Published:** 2021-07

**Authors:** Bethany Little, Ossama Alshabrawy, Daniel Stow, I. Nicol Ferrier, Roisin McNaney, Daniel G. Jackson, Karim Ladha, Cassim Ladha, Thomas Ploetz, Jaume Bacardit, Patrick Olivier, Peter Gallagher, John T. O'Brien

**Affiliations:** 1 Institute of Neuroscience, Newcastle University, Newcastle upon Tyne, UK; 2 Interdisciplinary Computing and Complex BioSystems (ICOS) group, School of Computing, Newcastle University, Newcastle upon Tyne, UK; 3 Faculty of Science, Damietta University, New Damietta, Egypt; 4 Institute of Health and Society, Newcastle University, Newcastle upon Tyne, UK; 5 Faculty of Engineering, Bristol University, Bristol, UK; 6 Open Lab, School of Computing, Newcastle University, Newcastle upon Tyne, UK; 7 Cascom Ltd, Newcastle upon Tyne, UK; 8 School of Interactive Computing, Georgia Institute of Technology, Atlanta, GA, USA; 9 Faculty of Information Technology, Monash University, Melbourne, Australia; 10 Department of Psychiatry, University of Cambridge, Cambridge, UK

**Keywords:** Ageing, deep learning, late-life depression, social functioning, speech, wearable technology

## Abstract

**Background:**

Late-life depression (LLD) is associated with poor social functioning. However, previous research uses bias-prone self-report scales to measure social functioning and a more objective measure is lacking. We tested a novel wearable device to measure speech that participants encounter as an indicator of social interaction.

**Methods:**

Twenty nine participants with LLD and 29 age-matched controls wore a wrist-worn device continuously for seven days, which recorded their acoustic environment. Acoustic data were automatically analysed using deep learning models that had been developed and validated on an independent speech dataset. Total speech activity and the proportion of speech produced by the device wearer were both detected whilst maintaining participants' privacy. Participants underwent a neuropsychological test battery and clinical and self-report scales to measure severity of depression, general and social functioning.

**Results:**

Compared to controls, participants with LLD showed poorer self-reported social and general functioning. Total speech activity was much lower for participants with LLD than controls, with no overlap between groups. The proportion of speech produced by the participants was smaller for LLD than controls. In LLD, both speech measures correlated with attention and psychomotor speed performance but not with depression severity or self-reported social functioning.

**Conclusions:**

Using this device, LLD was associated with lower levels of speech than controls and speech activity was related to psychomotor retardation. We have demonstrated that speech activity measured by wearable technology differentiated LLD from controls with high precision and, in this study, provided an objective measure of an aspect of real-world social functioning in LLD.

## Introduction

Late-life depression (LLD) is a common disorder associated with pervasive impairments in daily functioning (Fiske, Wetherell, & Gatz, 2009). Compared to depression in younger adults, LLD is associated with an increased burden of physical illness, more impaired functioning, more severe neuropsychological impairment, particularly in executive and psychomotor functioning and a poorer clinical outcome (Fiske et al., 2009; Thomas et al., 2009). Compared to healthy controls, LLD is associated with reduced social functioning, including lower social activity and social integration, lower instrumental and emotional support, smaller social networks and poorer quality of relationships (Chao, 2011; Mechakra-Tahiri, Zuzunegui, Preville, & Dube, 2009; Santini, Koyanagi, Tyrovolas, Mason, & Haro, 2015). Social functioning appears to have an important role in illness onset, course and outcome (Schwarzbach, Luppa, Forstmeier, König, & Riedel-Heller, 2014).

Social functioning is typically measured by patient or carer self-report, which is prone to error and biases from memory, mood and cognition (Hodgetts, Gallagher, Stow, Ferrier, & O'Brien, 2017). Since depression is associated with a negative bias in memory and cognition (Romero, Sanchez, & Vazquez, 2014), and since memory typically declines with age (Thomas et al., 2009), it is likely that self-report measures from patients with LLD are particularly prone to these biases. Further, the various published methods on social functioning in depression are heterogeneous and often measure different aspects of social functioning that are independent and difficult to compare (Santini et al., 2015). Thus, more objective, replicable measures of social functioning in LLD are needed.

Previous research has demonstrated the utility of wearable technology (e.g. actigraphs) to objectively measure physical activity in participants with LLD, with these methods producing more accurate measures than self-report scales (O'Brien et al., 2017; Prince et al., 2008). Consequently, it has been suggested that wearable technology could be useful in more objectively quantifying social activity in participants with LLD and, specifically, that wearable devices could detect speech activity that an individual is exposed to and engages in, as an ecologically valid measure of social interaction (Hodgetts et al., 2017). The continuous monitoring of daily functioning in participants' natural environment would facilitate automated transmission and analysis of data, providing a more timely and accurate assessment of depressive symptoms. Such improvements in assessment could help alleviate the large social and economic impact of depression (Hirschfeld et al., 2000; Kessler et al., 2003).

Depression is associated with atypical language patterns, such as more single-clause sentences, incomplete phrases and reduced utterances (Smirnova et al., 2018; Tackman et al., 2019). Patients with depression show quieter speech, reduced variation of volume and pitch and reduced prosody (Alpert, Pouget, & Silva, 2001; Yang, Fairbairn, & Cohn, 2013). Listeners who were naïve to the depressive state of a speaker can perceive the severity of depression from vocal recordings of people with depression (Yang et al., 2013). Changes in depressive symptoms are associated with differences in speech patterns and features (Cummins, Sethu, Epps, Schnieder, & Krajewski, 2015; Mundt, Vogel, Feltner, & Lenderking, 2012), and depression-related speech features can be found across different languages (Özkanca, Demiroglu, Besirli, & Celik, 2018). Such abnormal speech is thought to be related to psychomotor retardation in depression, a central feature of the disorder (Flint, Black, Campbell-Taylor, Gailey, & Levinton, 1993; Quatieri & Malyska, 2012; Scherer, Lucas, Gratch, Rizzo, & Morency, 2016). Speech could therefore be a key component in developing an accurate biomarker for depression and there has been recent interest in analysing depressed speech automatically (He & Cao, 2018; Jiang et al., 2018; Li, Fu, Shao, & Shang, 2018; Williamson et al., 2019). Automated analyses of specific acoustic features of speech can distinguish participants with depression from controls with accuracy levels of 75–80%, with the former showing shortened voice onset time, decreased second formant transition and increased spirantisation (Flint et al., 1993; Jiang et al., 2017; Scibelli et al., 2018; Yang et al., 2013). Acoustic speech analysis has been used to predict depression in at-risk participants 2 years before diagnosis with up to 74% accuracy (Ooi, Lech, & Allen, 2014). Similarly, automated analysis of language features can differentiate patients with schizophrenia and bipolar disorder from controls with 96% accuracy (Voleti et al., 2019).

Most of the studies to date measure speech in controlled settings (e.g. recording participants reading passages aloud in quiet rooms) and focus on detecting specific features of speech (He & Cao, 2018; Jiang et al., 2018; Li et al., 2018). An alternative approach would be to use wearable devices to objectively detect how much speech participants encounter and produce in their natural environment. Detecting speech this way could serve as a proxy for social interaction, encompassing numerous factors of social functioning that are often independently measured with different self-report scales (Santini et al., 2015). The recognition rate of depression has been shown to be higher in spontaneous speech compared to read speech (Alghowinem et al., 2013). Recent advances in technology, such as deep learning based speech detection, allow the accurate detection and analysis of speech in a way that protects the privacy of all participants (Cummins, Baird, & Schuller, 2018).

We tested the utility of a novel wrist-worn device and deep learning algorithms to detect speech as an objective indicator of social interaction in LLD and healthy controls. This programme of research had two main aims: the development and evaluation of the methodology and the application of the optimal method to explore its utility in older adults with and without depression. Only details of the latter are reported here. Our primary hypothesis was that LLD would show a lower mean level of total speech detected than controls. We also predicted that, out of all speech detected, LLD would produce a smaller proportion of speech themselves, compared to controls. As exploratory hypotheses, we tested whether groups differed in speech activity at different times of day and investigated whether speech would correlate with self-reported social functioning, severity of depression, cognitive functioning and motor activity.

## Methods

### Participants

Twenty-nine community-dwelling participants aged >60 with current major depression were recruited from secondary care services in the North East of England. Depression was diagnosed using DSM-IV criteria, as assessed by the Mini-International Neuropsychiatric Interview (MINI). Twenty-nine aged-matched healthy controls with no history of depression (self-report) or current depression (MINI) were recruited from a local volunteer database. Exclusion criteria for both groups included: severe or unstable physical illness (e.g. recent cardiac events, diabetes and cancer); cognitive impairment or dementia; Mini Mental State Examination (MMSE) score <24; acquired brain injury or stroke; recent history or current substance abuse; uncorrected visual or auditory deficits and history of electroconvulsive therapy (<6 months for LLD, any history for controls). All participants had English as a first language. The study was approved by the National Research Ethics Service Committee for the North East of England. Written informed consent was obtained from each participant after the procedure had been fully explained.

### Materials and measures

#### The wearable device

**Fig. 1.**
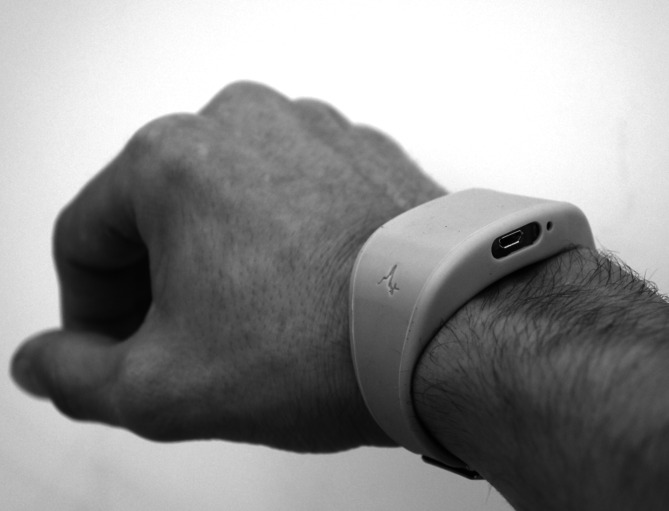
The wearable device.

The acoustic environment was measured using a custom-designed wrist-mounted device (Fig. 1; device repository available at www.github.com/digitalinteraction/openmovement). The device also measured physical activity, which we reported previously (O'Brien et al., 2017). The device incorporated a lithium ion battery, solid-state memory, a tri-axial accelerometer and a low fidelity (mono 8 kHz) microphone. All components, including internal storage, were encased in a thermoplastic cover. An injected resin compound ensured water-resistance. The device was attached to the wrist using a custom-designed, adjustable, hypoallergenic silicone band.

#### Clinical, functional and social assessments

The Montgomery-Asberg Depression Rating Scale (MADRS) and the 15-item Geriatric Depression Scale (GDS-15) measured severity of depression (Montgomery & Asberg, 1979; Sheikh & Yesavage, 1986). Short-Form Health Survey (SF-36) measured overall health and quality of life (Ware & Sherbourne, 1992). The Instrumental Activities of Daily Living (IADL) Scale measured ADL (Lawton & Brody, 1969). Social support, social network and loneliness were measured using the Duke Social Support Index (DSSI), the Lubben Social Network Scale-Revised (LSNS-R) and the UCLA Loneliness Scale (UCLA-LS; 10-item version), respectively (George, Blazer, Hughes, & Fowler, 1989; Knight, Chisholm, Marsh, & Godfrey, 1988; Lubben, Gironda, & Lee, 2002). These scales were chosen to measure social functioning on the basis of a previous review (Hodgetts et al., 2017).

#### Neuropsychological assessment

Cognitive ability was assessed with a comprehensive neuropsychological assessment reported previously (O'Brien et al., 2017), consisting of: Digit Span Forwards and Backwards, Digit Symbol Substitution Task (DSST), a facial emotion processing task (FERT), Trail Making Task A and B, Rey Auditory Verbal Learning task, FAS verbal fluency task and the Rivermead Behavioural Memory Test (Adams et al., 2016; Strauss, Sherman, & Spreen, 2006). Also included were four tasks from the Cambridge Neuropsychological Test Automated Battery (CANTAB): paired associates learning, spatial span, spatial working memory and affective go/no-go. The National Adult Reading Test (NART) estimated premorbid intelligence. Tasks were administered according to standardised instructions and manuals. All tasks were pen-and-paper, except CANTAB and FERT, which were carried out on a laptop with a 12.5-inch colour touchscreen and keyboard.

### Procedure

A baseline assessment involved collection of demographic information, self-report of medication, physical and mental health and completion of the MINI, MMSE, MADRS, GDS-15, NART, Digit Span, DSST and FERT. Three home visits then took place: on day one, the device was fitted and SF-36, IADL, DSSI, LSNS-R and UCLA-LS were conducted. Since the device battery lasted for less than 7 days, a second visit occurred between days two and six, when the initial device was swapped for a fully charged device. After seven days, the device was collected and remaining cognitive tasks were completed.

### Analysis of speech data

We developed two deep learning models to detect speech. The first model classified speech *v.* non-speech using the whole acoustic recording. The second model classified speech produced by the wearer (i.e. participant) *v.* speech of others, using the acoustic data that were originally classified as speech by the first model. Both classifiers were blind to the group status of each participant and this information was never used as part of each training process. Our methods of automatic analysis allowed speech to be objectively detected while maintaining the privacy of participants. We previously reported a high level of compliance with the device protocol (92% for each group; O'Brien et al., 2017).

#### Classifying speech *v.* non-speech

Device changeover days were ‘stitched’ together to form a single day. Acoustic data were pre-processed by uniformly rescaling the speech signals to the range (−1,1) and then split into frames of 32 ms length. The frames were normalised (zero mean and unit variance) and fed into our deep learning architecture for speech prediction in naturalistic environments (see online Supplementary Textbox S1 for details).

The classifier was trained using an independent set of acoustic recordings (training dataset) that were previously created from a separate group of healthy controls in a pilot study (*N* = 15; ~20 h in total). Pilot participants wore the device in a variety of settings in which naturalistic speech can occur (e.g. indoors, outdoors, in busy shopping centres) and consented to the research team listening to the recordings so that they could be annotated to denote segments of speech and non-speech. This allowed the predictive performance of the classifier to be evaluated. The evaluation was done using Leave One Session Out cross-validation, where we left one of the recordings out for validation and trained a model with all the others. The resulting model could classify speech in these recordings with an accuracy of 93.8% (sensitivity 94.6% and specificity 87.4%). Online Supplementary Figs S1 and S2 illustrate the technical process.

The classifier developed on the training dataset was then applied to the recordings of the current sample. The classifier detected any speech in the environment, i.e. it did not discriminate participants' speech from the speech of other people. It was trained to exclude speech from other sources such as television, radio and any other device-generated speech. Therefore, our measure of speech reflects the speech of all humans in the environment.

The output of the classifier was the probability of speech being detected in each processed frame. Each minute was considered to contain speech if the average probability of its frames was above a threshold of 0.5. For each day of recording, the number of minutes of speech was divided by the total number of minutes in that epoch (i.e. 1440 for 24 h), to produce a percentage of speech for that day. The average percentage for 7 days was then calculated for each participant. The average percentage of speech was also calculated for morning (6 am–12 pm), afternoon (12 pm–6 pm) and evening (6 pm–12 am) periods in the same way.

#### Classifying wearer speech *v.* other speech

A second deep learning model was developed using the training dataset to differentiate the wearer's speech from the speech of others. This model followed the same pre-processing procedure as the previous model with a different architecture (see online Supplementary Textbox S1). The same evaluation method was used; this model achieved an accuracy of 89.95% (sensitivity 90.3% and specificity 86.2%).

The trained classifier was applied to the minutes of speech classified by the first model (i.e. excluding data that was previously classified as non-speech). The output was the probability of wearer's speech being detected in each speech frame. We calculated the percentage of wearer speech in each minute by counting frames considered as wearer speech (i.e. probability >0.5) and dividing by the total number of speech frames in that minute. We then averaged this per-minute value across all speech minutes for each participant. This resulted in an average percentage of speech that was produced by the wearer, out of all data that was initially classified as speech.

Outputs from the two models are not directly comparable: since the input to the models differ (all frames *v.* speech frames only), they require different procedures to compute the measures. We compared the performance of our model on the discussion dataset against the performance of a variety of existing methods used for voice activity detection (see online Supplementary textbox S1 for details) and found that our model resulted in the highest performance evaluation (F1) measure.

### Statistical analysis

Scores from neuropsychological tests were standardised based on control group mean and standard deviation and organised into five cognitive domains: Executive Working Memory; Attention and Psychomotor Speed; Short-Term Memory; General Memory; Emotional Processing and Grand cognitive score (as reported previously (O'Brien et al., 2017); see online Supplementary Textbox S2). Group differences on all variables were assessed using two-tailed independent *t* tests; Mann–Whitney *U* tests were used for skewed data. Two-tailed Pearson's correlations were used to test linear relationships between speech measures and key variables; Spearman's rank order correlations were used for skewed data.

## Results

Table 1 displays group demographics, clinical characteristics, self-reported social functioning, speech data and group differences. Groups did not differ in sex, living status, handedness, age or premorbid IQ. LLD had fewer years of education and lower MMSE scores than controls. LLD scored higher than controls on UCLA-LS, reflecting higher self-reported loneliness, and on both depression scales (MADRS and GDS-15). LLD scored lower than controls on general health and functioning (SF-36 and IADL), and self-reported social interaction and social network (DSSI and LSNS-R). We reported neuropsychological scores previously: after NART IQ was added to the model as a covariate, LLD showed significantly poorer performance compared to controls on domains of Executive Working Memory, Attention and Psychomotor Speed, General Memory and grand cognitive performance (O'Brien et al., 2017). Given that groups differed in years of education, we repeated this analysis after adding education to the model as a covariate and the results were the same (see online Supplementary Table S1). Since our a priori predictions did not include this variable, we focus on analysis without controlling for education.

**Table 1.** Demographic information, clinical and social characteristics, speech measures and group comparisons

	LLD			Healthy controls			Group differences			
	Mean	s.d.	*N*	Mean	s.d.	*n*	Statistic	df	*p*	Effect size
Demographics										
Female	72%		21/29	76%		22/29	χ^2^ = 0.090	1	0.764	*Φ* = 0.039
Lives alone	59%		17/29	38%		11/29	χ^2^ = 2.486	1	0.115	*Φ* = 0.207
Age in years	74.2	6.0	29	74.0	5.9	29	*t* = −0.110	56	0.913	*d* = 0.034
NART score	109.0	9.9	29	114.2	10.3	29	*t* = 1.962	56	0.055	*d* = 0.515
Education in years	10.9	2.2	29	13.3	3.3	29	*U* = 254.0, *z* = −2.634		0.008*	*η*^2^ = 0.120
MMSE score	27.7	1.8	29	28.8	1.1	29	*U* = 270.5, *z* = −2.548		0.014*	*η*^2^ = 0.112
Clinical and social characteristics										
MADRS	28.3	8.6	28	1.0	1.6	29	*U* = 812.0, *z* = 6.589		<0.001*	*η*^2^ = 0.762
GDS-15	9.7	3.1	28	0.9	1.4	29	*U* = 803.0, *z* = 6.434		<0.001*	*η*^2^ = 0.726
SF-36 total	38.3	19.9	29	78.7	10.5	29	*t* = 9.661	42.44	<0.001*	*d* = 2.539
IADL total	6.9	1.7	29	8.0	0.2	29	*U* = 227.5, *z* = −3.905		<0.001*	*η*^2^ = 0.263
DSSI total social support	41.7	5.2	29	48.4	6.3	29	*U* = 131.0, *z* = −4.515		<0.001*	*η*^2^ = 0.351
LSNS-R total	25.5	7.6	29	36.8	7.4	29	*U* = 123.5, *z* = −4.625		<0.001*	*η*^2^ = 0.369
UCLA-LS total	18.5	7.3	29	5.8	4.6	29	*U* = 765.0, *z* = 5.366		<0.001*	*η*^2^ = 0.496
Speech										
Proportion of speech detected 24-h	0.02	0.01	29	0.13	0.03	29	*U* = 0.000, *z* = −6.541		<0.001*	*η*^2^ = 0.738
Proportion of speech detected morning	0.04	0.02	29	0.24	0.04	29	*t* = 19.980	39.82	<0.001*	*d* = 5.250
Proportion of speech detected afternoon	0.03	0.02	29	0.24	0.05	29	*U* = 0.000, *z* = −6.540		<0.001*	*η*^2^ = 0.737
Proportion of speech detected evening	0.01	0.01	29	0.04	0.02	29	*t* = 8.585	31.05	<0.001*	*d* = 2.259
Proportion of speech produced by wearer	0.03	0.003	29	0.11	0.01	29	*t* = 35.037	32.48	<0.001*	*d* = 10.127

LLD, Late-Life Depression; s.d., Standard Deviation; df, Degrees of Freedom; NART, National Adult Reading Test; MMSE, Mini Mental State Exam; MADRS, Montgomery-Asberg Depression Rating Scale; GDS-15, Geriatric Depression Scale; SF-36, Short-Form Health Survey; IADL, Instrumental Activities of Daily Living; DSSI, Duke Social Support Index; LSNS-R, Lubben Social Network Scale-Revised; UCLA-LS, UCLA Loneliness Scale.

*Note*: *Significant at 0.05 level.

**Fig. 2.**
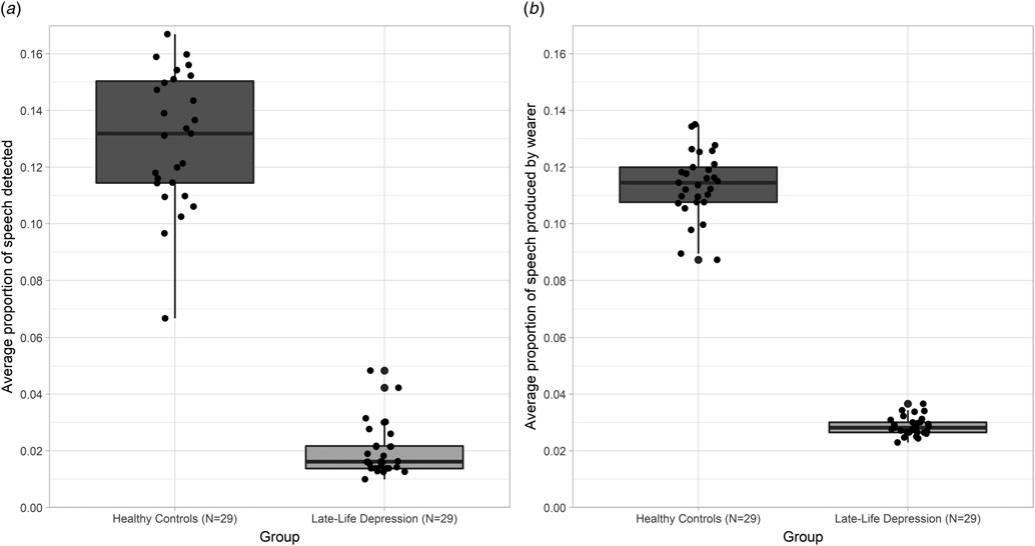
(a) Mean proportion of speech detected in a 24-h period (averaged over 7 days) and (b) mean proportion of speech produced by the wearer themselves (out of all speech detected) for LLD and healthy controls. Dots represent individual participants and are randomly spread across the *x*-axis within each group. Groups differed significantly in the proportion of speech detected in 24 h, such that all participants with LLD showed lower levels of speech detected than all healthy controls (*U* = 0.0, *z* = −6.541, *p* < 0.001). Of all speech detected, LLD produce a smaller proportion of speech themselves, compared to healthy controls (*t*_(32.477)_ = 38.562, *p* < 0.001).

Figure 2 illustrates the speech data for each group. Groups differed in average speech activity over a 24-h period, *U* = 0.0, *z* = −6.541, *p* < 0.001. On average, speech was detected for 2% (±1%) of the day in LLD, whereas in controls, speech was detected for 13% (±3%) of the day. This difference was highly significant and strikingly there was no overlap between groups. Groups also differed in the proportion of speech they produced themselves out of all speech detected, *t*_(32.477)_ = 38.562, *p* < 0.001. In the LLD group, 3% (±0.3%) of all speech detected was produced by the wearer, whereas, in the control group, 11% (±1%) of all speech detected was produced by the wearer.

**Fig. 3.**
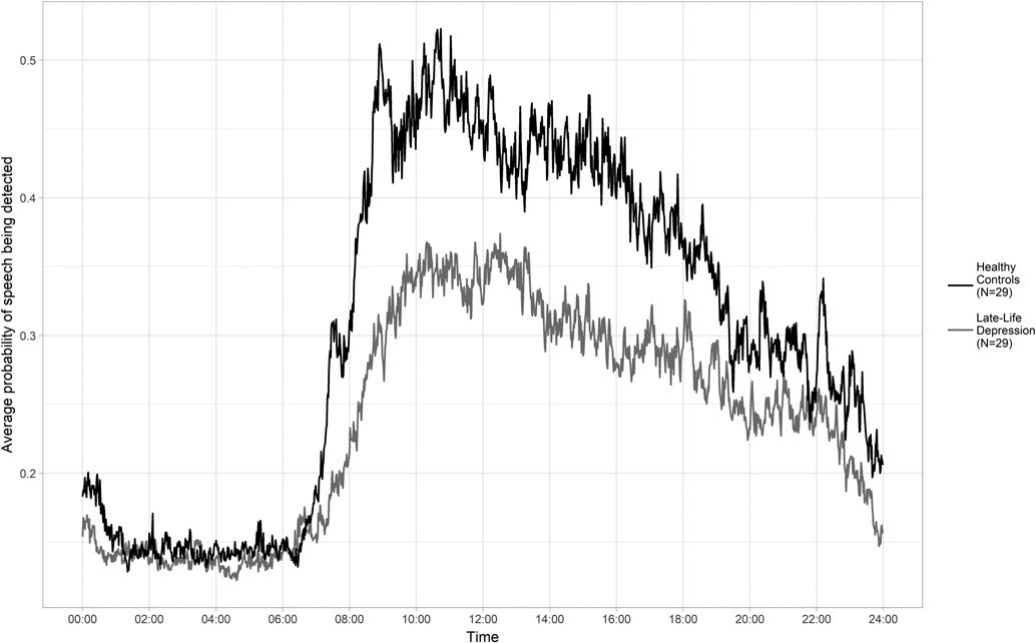
Mean probability of speech being detected for participants with LLD and healthy controls across a 24-h period (averaged over 7 days).

**Fig. 4.**
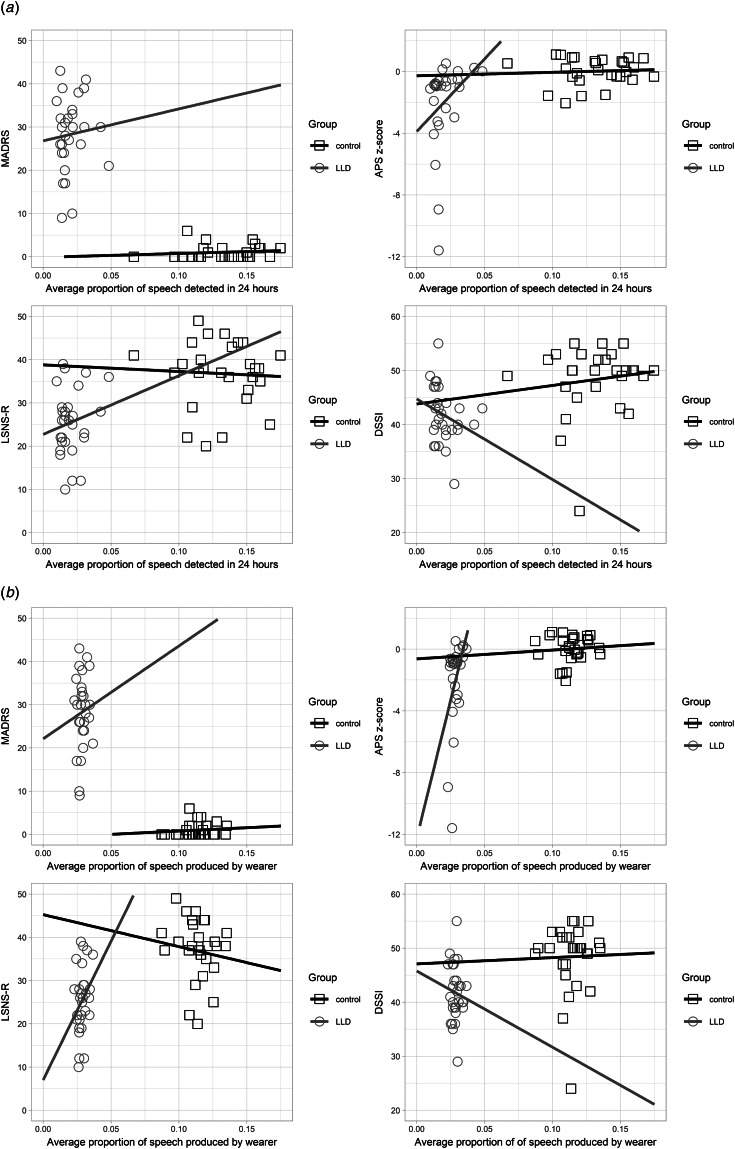
Relationships between key variables and: (a) mean proportion of total speech detected across 24-h (averaged over 7 days); and (b) mean proportion of speech produced by the wearer (out of all speech detected), for participants with LLD (*N* = 29) and healthy controls (*N* = 29). MADRS, Montgomery-Asberg Depression Rating Scale; APS, Attention and Psychomotor Speed; DSSI, Duke Social Support Index; LSNS-R, Lubben Social Network Scale-Revised.

Figure 3 shows the mean speech activity levels for LLD and control groups over a 24-h period. Groups differed in the proportion of speech detected at each time of day (morning, afternoon and evening; see Table 1). Figure 4 displays correlations of each speech measure with key variables for each group. For LLD, both the proportion of all speech detected and the proportion of speech produced by the wearer were significantly correlated with Attention and Psychomotor Speed (*r*_s_(27) = 0.428, *p* = 0.021 and *r*_s_(27) = 0.474, *p* = 0.009, respectively), where more speech detected was associated with a higher Attention and Psychomotor Speed score. No other correlation was significant (see online Supplementary Table S2). In exploratory analysis, neither of the two speech measures correlated with any of the movement measures in LLD, but all correlations between speech and movement measures were significant in the control group (see online Supplementary Table S3).

## Discussion

This study is the first to utilise a novel wearable device to objectively detect speech in the naturalistic environment of participants with LLD and healthy controls over a 7-day period. The initial speech activity measure, which was developed on an independent training dataset, differentiated LLD and controls with 100% accuracy, with speech detection in LLD being greatly diminished compared to controls. This difference was apparent across the course of the day. The second speech activity measure, which detected the device wearer's speech specifically, showed that, out of all data that was initially classified as speech, LLD participants spoke much less than controls, and also differentiated groups with 100% accuracy. Cognitive performance and self-reported social and general functioning were lower in LLD than in controls, in line with previous research (Fiske et al., 2009; Thomas et al., 2009).

Exploratory analysis revealed that the percentage of speech detected in a 24-h period and the percentage of speech produced by the wearer were both associated with attention and psychomotor speed in the LLD group. Considering that abnormal speech in depression has been linked to psychomotor retardation, a central feature of the disorder (Flint et al., 1993; Quatieri & Malyska, 2012), these results could be interpreted as some support for the development of speech measures as a biomarker for depression. However, further validation of this is needed, since we did not correct for multiple comparisons in the exploratory analysis. Speech activity and motor activity were not correlated in the LLD group, which may be expected because of the particularly marked reduction in speech that was seen in this group.

Since participants with LLD and controls differed so markedly in speech activity that they encountered and speech that they produced, it is perhaps surprising that speech activity did not correlate with the clinical scales of depression in the LLD group. Similarly, it is unexpected that speech activity did not correlate with the self-report scales of social functioning. It could be that our measures of speech reflect a more accurate measure of social interaction than the self-report scales, which are influenced by bias. Indeed, previous research has highlighted that a discrepancy between subjective and objective measures of social functioning may be due to a bias towards pessimism in participants with depression (Santini et al., 2015). Discrepancies between objective and self-report measures of physical activity have also been found (Prince et al., 2008). These results could also be explained by a floor effect in the speech data of the LLD group: there may have been insufficient variation to produce significant correlations. It is also possible that these measures of speech represent a depression-related construct that is independent of any of the other variables measured and that is not included in either depression scale.

Another consideration is whether lower speech activity reflects the current depressive state or whether it reflects something that distinguishes those who are prone to depression from those who are not (i.e. depressive trait). Previous research suggests that changes in some aspects of speech patterns have been found to be related to changes in the depressed state in participants with depression, while others are related to a depressive trait (Alpert et al., 2001; Mundt et al., 2012). If our speech measures reflect a trait of LLD, this may explain why speech did not correlate with MADRS or GDS-15, which measure the depressive state.

Limitations to our study include cross-sectional design and small sample sizes. While the classifier was accurate in detecting speech and non-speech in the training dataset, which consisted of healthy controls, we could not directly generate the accuracy of the classifier with the study participants' data since listening to and annotating the recordings was not ethically possible. Therefore, we cannot conclude exactly how accurate the speech measures are for people with LLD. Since depression has been associated with abnormalities in specific acoustic features of speech and depressed speech appears to contain more noise (Alpert et al., 2001; Flint et al., 1993; Taguchi et al., 2018), it is possible that the classifier may perform differently with the LLD group than controls. This requires further investigation and future research should validate measures of speech by comparing the output of different speech classifiers in patients with LLD.

Since groups did not differ in living status, we did not control for this in our analysis. Some studies suggest that living status can predict depression, while others suggest it is unrelated to depressive symptoms (Alexandrino-Silva, Alves, Tófoli, Wang, & Andrade, 2011; Schwarzbach et al., 2014). This factor may be particularly important with our measure of speech, since living alone may directly influence the speech activity detected. Other factors that we did not control for that may influence the association between social functioning and depression include gender, culture, socio-economic status and whether participants live in rural, urban or metropolitan areas (Jiang et al., 2017; Mechakra-Tahiri et al., 2009; Santini et al., 2015; Schwarzbach et al., 2014). Similarly, we did not take into account whether LLD was early-onset or late-onset; these appear to be two distinct types of LLD that may have different associations with social functioning (Sachs-Ericsson et al., 2012).

Our objective speech measures do not capture qualitative or subjective factors of social interaction, such as satisfaction with social support, which have been shown to be powerful, consistent predictors of depression in older people (Chao, 2011; Schwarzbach et al., 2014). Neither do they discriminate the type of social interactions that may be important in LLD, such as emotional and instrumental support. Measuring speech also has pragmatic limitations, as it excludes people with verbal communication difficulties. Finally, this measure may vary in accuracy for different cohorts, due to changes in the way people socialise and communicate (i.e. verbally *v.* non-verbally via technology).

Nevertheless, the methods presented here can accurately distinguish depressed participants from controls and may be a useful marker for LLD. A particular strength of the study was that the device was unobtrusive and we found high adherence with wearing the device (O'Brien et al., 2017), demonstrating the feasibility of using such devices with older participants. If developed further, this measure has the potential to be used in screening for LLD, facilitating early diagnosis, and has implications for monitoring long-term health and recovery. The methods presented here provide a starting point for further research using raw sensor recordings and automatic analysis to investigate speech and social functioning in LLD.

Future research should replicate our findings to test external validity and should control for potential confounds such as living status, gender and culture. Further research is needed to investigate whether this measure reflects social functioning, as we intended, or whether it captures another LLD-related factor. It would also be of interest to investigate whether speech activity detected reflects a trait marker of the depression or current depressive state. Longitudinal research should measure changes in speech over the onset, course and remission of depression, and investigate causality and the direction of the relationship between speech and LLD. Methods of detecting more specific variables from this speech data should also be developed, such as measuring acoustic characteristics of the wearer's speech (e.g. prosody) and modelling the wearer's speech against the speech of other people. The development of multi-modal assessments, for example, analysing speech and movement characteristics together should be developed to produce a more holistic and ecologically valid measure of daily functioning in LLD.

## Supplementary material

For supplementary material accompanying this paper visit https://doi.org/10.1017/S0033291719003994. click here to view supplementary material
