# Supplementary material for: Deep learning-based automated speech detection as a marker of social functioning in late-life depression
Source: Psychol Med. 2020 Jan 16;51(9):1441–50. doi: 10.1017/S0033291719003994 (PMC8311821; doi:10.1017/S0033291719003994)
Supplement: Supplementary file 1 [file S0033291719003994sup001.docx]

# Supplementary Materials

**Supplementary Textbox 1.** Details of both deep learning architectures.

**Details of our deep learning architecture to classify speech vs non-speech in naturalistic environments**

This deep learning model is a deep bi-directional long short-term memory (BLSTM); a combination of bi-directional recurrent neural network (BRNN; 1) and long short-term memory (LSTM; 2) units. LSTM units are widely used to process temporal data such as sound (3). The architecture of the model consists of eight layers of BLSTM, followed by six layers of LSTMs, four fully-connected layers, and softmax layer. All LSTM layers use the hyperbolic tangent as activation function. Three of the fully-connected layers use Maxout and the fourth uses the ReLU activation function. All the BLSTM and LSTM layers are trained using the Adam gradient descent algorithm (4). All the fully-connected layers are trained using Rmsprop algorithm (5). The learning rate used is 10-5, and the regularisers used are dropout, batch normalisation, and l2 regularization. We implemented this network using the DeepLearning4J framework (https://deeplearning4j.org). Manual feature engineering processing was not required.

**Details of our deep learning architecture to classify wearer’s speech vs other speech in naturalistic environments**

This deep learning model starts with Convolutional Autoencoder (Encoder: 3 convolution layers, 3 MaxPooling layers and one fully-connected layer; Decoder: 3 convolution layers, 3 up-sampling layers and one fully-connected layer) followed by 3 layers of BLSTM, then 4 layers of LSTMs, and finally the softmax layer. All LSTM layers use the hyperbolic tangent as the activation function. The training algorithm for all layers is the Adam algorithm (4). The learning rate used is 10-5, and the regularisers used are dropout, batch normalisation, and l2 regularization. The classifier is trained only on the wearer speech and speech of other speakers from the training dataset (i.e. non-speech was not included in the training).

The source code for both models is available at https://github.com/OssamaAlshabrawy/NatSpeech

**Comparison of our deep learning architecture to other voice activity detection methods**

The performance of our speech activity detection method was compared against a variety of existing voice activity detection methods, ranging from classic methods such as Sohn99 (6), Random Forest, Support vector Machines, Ramirez05 (7) to more recent methods such as Ying11 (8), Multi-resolution stacking (9), Sehgal18 (10), Gelly18 (11); the latter three are based on deep learning. The first set of experiments were conducted on public datasets: Aurora 2 (12), Aurora 4 (13) and TIMIT (14). On the discussion dataset, we compared our measure with Sehgal18 and Gelly18 because they were found to be the best methods in the initial experiments. Our model resulted in the highest performance evaluation (F1) measure.

**References**

1. **Scibelli F, Roffo G, Tayarani M, et al.** (2018). Depression Speaks: Automatic discrimination between depressed and non-depressed speakers based on non-verbal speech features, in *IEEE International Conference on Acoustics, Speech and Signal Processing (ICASSP)*, pp 6842–6846.
2. **Hochreiter S, Schmidhuber J** (1997). Long short-term memory. *Neural Computation* **9**, 1735–1780.
3. **Medennikov I, Bulusheva A** (2016). LSTM-Based Language Models for Spontaneous Speech Recognition, in *International Conference on Speech and Computer*, pp 469–475.
4. **Kingma DP, Ba J** (2014). Adam: A Method for Stochastic Optimization, *inarXiv preprint arXiv:1412.6980*
5. **Hinton G, Deng L, Yu D, et al.** (2012). Deep neural networks for acoustic modeling in speech recognition. *IEEE Signal Process Mag* **29**, 82–97
6. **Sohn J, Kim NS, Sung W** (1999). A statistical model-based voice activity detection. *IEEE Signal Processing Letters* **6**, 1–3.
7. **Ramirez J, Segura JC, Bentitez C, Garcia L, Rubio A** (2005) Statistical voice activity detection using multiple observation likelihood ratio test. *IEEE Signal Processing Letters* **12**, 689-692.
8. **Ying D, Yan Y, Dang J, Soong F** (2011). Voice Activity Detection based on unsupervised learning framework. *IEEE Transactions on Audio, Speech and Language Processing* **19**, 2624–2644.
9. **Zhang XL, Wang D** (2016). Boosting Contextual Information for Deep Neural Network Based Voice Activity Detection. *IEEE/ACM Transactions on Audio, Speech, and Language Processing* **24**, 252–264.
10. **Sehgal A, Kehtarnavaz N** (2018). A Convolutional Neural Network Smartphone App for Real-Time Voice Activity Detection. *IEEE Access* **6**, 9017–9026.
11. **Garofolo JS** (1993). TIMIT acoustic-phonetic continuous speech corpus ldc93s1*. Philadelphia: Linguistic Data Consortium.*
12. **Pearce D, Hirsch H** (2000). The AURORA Experimental Framework for the Performance Evaluation of Speech Recognition Systems under Noisy Conditions. in *PROC. ICSLP* **4**, 29–32.
13. **Pearce D, Picone J** (2002). Aurora Working Group: DSR font end LVCSR Evaluation AU/384/02. *Inst. for Signal and Inf. Process.*
14. **Gelly G, Gauvain JL** (2018). Optimisation of RNN-Based Speech Activity Detection. *IEEE/ACM Transactions on Audio, Speech and Language Processing* **26**, 646–656.

**Supplementary Textbox 2.** Details of neuropsychological domains.

**Calculation of neuropsychological domains**

Scores for each neuropsychological test were standardised by calculating a z-score based on control group mean and standard deviation. Where appropriate, scores were inverted such that a higher score reflected better performance. Individual neuropsychological tests were then organised into neuropsychological domains by averaging the z-scores of the following groups of tests:

- **Executive Working Memory** comprised Verbal Fluency total score, Digit Span Backwards total score, and Cambridge Neuropsychological Test Automated Battery (CANTAB) Spatial Working Memory between errors 4–10 boxes.
- **Attention and Psychomotor Speed** comprised Trails A total time and Digit Symbol Substitution Task total time.
- **Short-Term Memory** comprised Digit Span Forwards total score and span length from the CANTAB Spatial Span task.
- **General Memory** comprised Rey Auditory Verbal Learning task (RAVL) presentations 1–5 total, RAVL maximum delayed recall, CANTAB Paired Associates Learning total errors adjusted, and Rivermead Behavioural Memory Test standardised profile score.
- **Emotional Processing** comprised median latency of correct responses from the CANTAB Affective Go/No-go task and Facial Emotion Processing task proportion of correct to incorrect answers.
- A **Grand cognitive** score was calculated by summing individual z-scores of all neuropsychological tests.

**Supplementary Table 1.** Results of group comparisons of neuropsychological performance (z-scores) of participants with Late-Life Depression (N=29) and healthy controls (N=29), with NART IQ and years of education added to the Analysis of Covariance model as covariates.

|  | *F* | *Degrees of Freedom* | *p* | *η*_p_^2^ |
| --- | --- | --- | --- | --- |
| Executive working memory | 5.813 | 1, 54 | .019* | .097 |
| Attention and psychomotor Speed | 7.796 | 1, 54 | .007* | .126 |
| Short-term memory | 2.822 | 1, 54 | .099 | .050 |
| General memory | 11.328 | 1, 54 | .001* | .173 |
| Emotional processing | .039 | 1, 54 | .843 | .001 |
| Grand cognitive performance | 17.412 | 1, 54 | <.001* | .244 |

*significant at the .05 level

**Supplementary Table 2.** Results of correlations of key study variables with speech data for participants with Late-Life Depression (N=29).

|  | Average percentage of speech detected in 24-hour period | Average percentage of speech produced by the wearer (out of all speech detected) |
| --- | --- | --- |
| Montgomery-Asberg Depression Rating Scale | *r_s_*(26) = .092, *p* = .643 | *r*(26) = .081, *p* = .681 |
| Attention and Psychomotor Speed z-score | *r_s_*(27) = .428, *p* = .021* | *r_s_*(27) = .474, *p* = .009* |
| Duke Social Support Index | *r_s_*(27) = -.315, *p* = .096 | *r*(27) = -.087, *p* = .653 |
| Lubben Social Network Scale-Revised | *r_s_*(27) = .118, *p* = .542 | *r*(27) = .274, *p* = .151 |

*significant at the .05 level

**Supplementary Table 3.** Results of Pearson (*r*) and Spearman (*r*_s_) correlations of movement measures (physical activity, entropy, and jerk)^[[1]](#footnote-1)^ with speech data for participants with Late-Life Depression (N=29) and healthy controls (N=29).

|  | Late-Life Depression | | Healthy controls | |
| --- | --- | --- | --- | --- |
|  | **Average percentage of speech detected in 24-hour period** | **Average percentage of speech produced by the wearer (out of all speech detected)** | **Average percentage of speech detected in 24-hour period** | **Average percentage of speech produced by the wearer (out of all speech detected)** |
| Physical Activity | *r_s_*(27) = -.167, *p* = .387 | *r*(27) = -.032, *p* = .871 | *r*(27) = .418, *p* = .024* | *r*(27) = .525, *p* = .003* |
| Entropy | *r_s_*(27) = -.007, *p* = .972 | *r*(27) = .064, *p* = .740 | *r*(27) = .457, *p* = .013* | *r*(27) = .488, *p* = .007* |
| Jerk | *r_s_*(27) = -.050, *p* = .798 | *r*(27) = .026, *p* = .893 | *r*(27) = .410, *p* = .027* | *r*(27) = .479, *p* = .009* |

*significant at the .05 level.


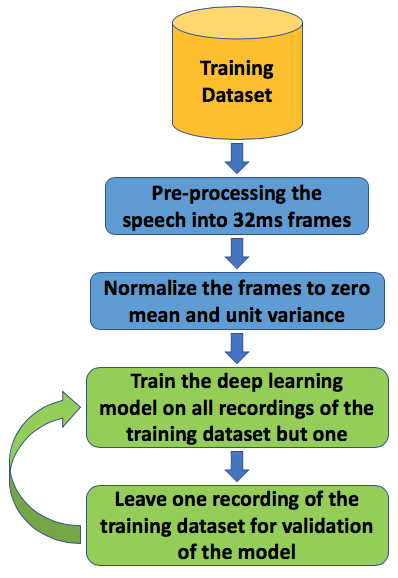


**Supplementary Figure 1.** Estimation of the predictive capacity of our deep learning speech prediction on the training dataset.


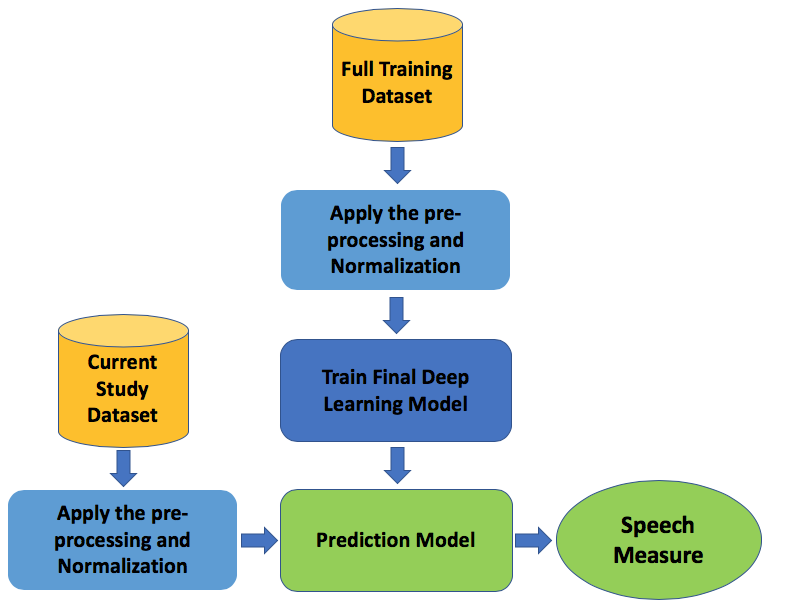


**Supplementary Figure 2.** Application of the speech prediction model to the recordings of the current study.

1. Group means and standard deviations of movement measures are reported previously: **O’Brien, Gallagher, Stow, Hammerla, Ploetz, Firbank, Ladha, Ladha, Jackson, McNaney, Ferrier & Olivier** (2017). A study of wrist-worn activity measurement as a potential real-world biomarker for late-life depression. *Psychological Medicine,* **47**, 93–102. [↑](#footnote-ref-1)
